# Understanding how and why travel mode changes: analysis of longitudinal qualitative interviews

**DOI:** 10.1186/s12966-024-01647-x

**Published:** 2024-09-02

**Authors:** Kate Garrott, Louise Foley, David Ogilvie, Jenna Panter

**Affiliations:** 1 MRC Epidemiology Unit, University of Cambridge, Cambridge, UK; 2 Institute of Applied Health Research, University of Birmingham, Birmingham, UK

**Keywords:** Active travel, Modal shift, Interviews, Qualitative

## Abstract

**Background:**

Encouraging alternatives to the car such as walking, cycling or public transport is a key cross-sector policy priority to promote population and planetary health. Individual travel choices are shaped by individual and environmental contexts, and changes in these contexts – triggered by key events – can translate to changes in travel mode. Understanding how and why these changes happen can help uncover more generalisable findings to inform future intervention research. This study aimed to identify the mechanisms and contexts facilitating changes in travel mode.

**Methods:**

Prospective longitudinal qualitative cohort study utilising semi-structured interviews at baseline (in 2021), three- and six-month follow up. Participants were residents in a new town in Cambridgeshire, UK, where design principles to promote walking, cycling and public transport were used at the planning stage. At each interview, we followed a topic guide asking participants about previous and current travel patterns and future intentions. All interviews were audio recorded and transcribed. Data analysis used the framework approach based on realist evaluation principles identifying the context and mechanisms described by participants as leading to travel behaviour change.

**Results:**

We conducted 42 interviews with 16 participants and identified six mechanisms for changes in travel mode. These entailed increasing or reducing access, reliability and financial cost, improving convenience, increasing confidence and raising awareness. Participants described that these led to changes in travel mode in contexts where their existing travel mode had been disrupted, particularly in terms of reducing access or reliability or increasing cost, and where there were suitable alternative travel modes for their journey. Experiences of the new travel mode played a role in future travel intentions.

**Implications:**

Applying realist evaluation principles to identify common mechanisms for changes in travel mode has the potential to inform future intervention strategies. Future interventions using mechanisms that reduce access to, reduce reliability of, or increase the financial cost of car use may facilitate modal shift to walking, cycling and public transport when implemented in contexts where alternative travel modes are available and acceptable.

**Supplementary Information:**

The online version contains supplementary material available at 10.1186/s12966-024-01647-x.

## Introduction

Replacing car use with active travel and public transport could facilitate multiple favourable outcomes for public and planetary health [1, 2] including increasing physical activity [3], reducing sedentary behaviour [4] and improving air quality [5]. Despite these benefits, the private car remains the dominant transport mode in many countries [6, 7], and reducing car use is a shared cross-sectoral policy priority [5]. 

Individual travel choices are shaped by both individual and environmental context, and changes to these contexts can explain periods of stability or change in individual mobility [8]. Key events may change the context within which people make their travel choices, and may therefore lead to changes in the travel mode selected. Within the literature on mobility geographies, the term ‘key events’ embraces both life events (e.g. having a child, moving house or acquiring a driving licence) and exogenous interventions (e.g. the introduction of a new bus timetable) that change contexts and may consequently influence travel patterns [9]. 

Exogenous interventions may be introduced that change travel patterns, either as their deliberate intention or as an unintended consequence. Deliberate interventions are typically implemented by actors such as national or local governments or transport service providers, and may target individual behaviour or wider economic, physical and social conditions. These interventions take place within a dynamic wider social context in which life events [8, 10] or other changes in the environment may also affect travel choices. Some research has focused on estimating the effect of specific *types* of key events on travel behaviour, such as residential relocation or the introduction of cycle lanes. While this has provided insights useful for policy and practice, findings have been mixed across travel modes [8], population subgroups [11] and locations, and estimating the effects of these changes without also considering the mechanisms involved may limit our understanding of how change occurs and therefore of the transferability of the findings [12]. 

Understanding the mechanisms by which key events bring about changes in travel mode is central to the approach of realist evaluation, which aims to understand what works, in what circumstances and for whom and is often used to evaluate or synthesise evidence about complex interventions [13]. Within realist evaluation, the term *mechanisms* denotes explanatory accounts that include the resources provided by an intervention and the reasoning and responses of participants to the intervention [14]. Furthermore, this approach explicitly considers that differences in contexts (the conditions of the setting in which an event occurs) or in the characteristics of individuals exposed to events may lead to different outcomes [12]. Identifying the contexts, mechanisms and outcomes can help build an explanation of how interventions work to bring about their effects that is both more detailed and more generalisable [15]. Applying this approach beyond intervention evaluation to understand mechanisms of changes in travel behaviour arising from key events has potential to uncover useful insights that can be applied within interventions.

There is limited research explicitly using realist approaches to explore the underlying processes of how a broader range of key events (i.e. not limited to ‘interventions’) may change the context for travel behaviour and thereby bring about changes in travel mode. One longitudinal qualitative study explored changes in cycling over three years in Cycling Demonstration Towns (CDT) in England, where there were major investments in cycling infrastructure, training and marketing [16]. This study identified triggers arising from key events that led to changes in cycling behaviour, while personal history, intrinsic motivations and the existing environment were contextual aspects that influenced behaviour. The authors provide illustrative examples to explain the reasoning for cycling behaviour change [17]. A young cyclist getting their first job, cites financial (cycling is cheaper than the bus), safety (there is somewhere safe to keep the bike) and availability (no car available) reasoning as forming the mechanism for a change in travel patterns.

We hypothesise that elaborating this type of analysis to describe and synthesise mechanisms across participants exposed to a variety of key events may be helpful in understanding the potential of a broader range of policy strategies to promote alternatives to the car. In this exploratory study, we tested a novel approach using longitudinal qualitative interviews to explore changes in travel mode arising from a variety of key events. We applied principles of realist evaluation to understanding the mechanisms and contexts that facilitate these changes, with a view to informing more generalisable principles for intervention design. Our specific objectives were to identify common mechanisms arising from key events that lead to changes in context, and to understand the contextual conditions that facilitate changes in travel mode.

## Methods

### Study design

This was a prospective longitudinal qualitative cohort study nested within a randomised controlled trial (RCT) to assess the feasibility of financial incentives for promoting alternatives to the car, implemented in Northstowe, a new town in Cambridgeshire, United Kingdom [18]. Eligible individuals for the RCT were those aged over 16 years and living in a household in Northstowe that had not claimed financial incentives, meaning they were either unaware of the incentives or had previously chosen not to claim them. The RCT was conducted while covid-19 was circulating (October 2021- July 2022), which may have influenced participants’ travel patterns and their attitudes towards different travel modes. A subset of participants from the RCT was invited to complete remote semi-structured interviews at baseline and at three and six months post-baseline. Participants who completed at least one follow-up interview were eligible for this analysis. No additional incentives were provided to participate in the semi-structured interviews.

All participants gave written informed consent at the beginning of each interview. The School of Humanities and Social Sciences Research Ethics Committee, University of Cambridge provided ethical approval (HVS/2019/2778).

### Setting

Northstowe is a new town currently under construction eight miles north of Cambridge, United Kingdom. At the time of the study, 908 of the projected 10,000 new homes were occupied. The town received Healthy New Town funding [19] and is intended to provide a sustainable environment promoting health and wellbeing. During the study, all residents lived within one mile of a bus stop, providing a service to Cambridge City Centre within 20 min. At the time of the study, available public facilities were two schools, open spaces, children’s play parks and outdoor leisure facilities but no shops or other amenities.

### Sample recruitment and representativeness

From RCT participants, we purposively selected a sample of participants for invitation to interview based on treatment group allocation, age, car ownership and baseline travel patterns in order to understand a broad range of perspectives and experiences. There were no additional eligibility criteria to participate in the interviews. Interested participants responded and completed an online consent form before arranging an appropriate time for the interview. For those who completed baseline interviews, we repeated this process at three and six months post baseline.

### Data collection

We conducted semi-structured interviews following a flexibly applied topic guide (Supplementary material A). The topic guide covered impressions of living in Northstowe, experiences of the environment, current travel patterns and intentions to change. Open-ended questions were used to minimise the risk of social desirability bias. Three and six month interviews also discussed changes in travel since the previous interview. The interviews also explored the use of financial incentives, which were the subject of the RCT. KG conducted all interviews between November 2021 and July 2022 via video call or telephone due to the continued circulation of covid-19 and wrote detailed field notes. Each interview lasted between 18 and 70 min, and was audio recorded and transcribed verbatim by a third party.

### Data analysis

All transcripts were imported to NVivo software to facilitate and manage the coding process. We analysed the transcripts using a framework approach [20] based on pre-defined realist evaluation principles [21]. We coded key events, contexts, mechanisms and outcomes as defined in Table 1. We linked these codes to maintain individual configurations and wrote contextual descriptions for each participant. We did not code any data related to the use of financial incentives (the intervention being tested in the parent RCT). While only one participant reported a direct effect of incentives on travel mode, we cannot rule out that the intervention worked in more implicit ways, for example by signalling approval of alternative travel modes. We compared the emerging codes and developed an analytical framework which was refined throughout the coding process, until a final analytical framework was applied to all transcripts.

**Table 1:** Definitions used ^a^

Concept	Definition
Key event	*Change* to a setting (physical, social, political, fiscal or organisational) or individual circumstance that is unrelated to the financial incentives studied within the parent RCT. These include changes primarily intended to alter travel behaviour (e.g. a new bus timetable), and changes capable of altering travel behaviour but not primarily intended to do so (e.g. new local amenities, having a child)
Context	The physical, social, political, fiscal or organisational conditions of the setting in which the event occurs (e.g. the existing bus network) and/or the physical, social or political characteristics of the individual exposed to the event (e.g. bicycle ownership, ability to cycle)
Mechanism	The process by which a key event interacts with people in a particular context to lead to travel behaviour change, including the reasoning of how people or populations responded to the key event. This process may be observable or hidden.
Outcome	Any change in travel mode reported by a participant, including changes for a single journey or multiple ongoing changes

^a^ adapted from Panter et al. (2019)

We charted the data, maintaining the linked individual configurations. Each row in our data table represented a unique configuration and each column represented a participant, with its heading containing the individual’s contextual description. Where a configuration was reported by a participant, we summarised the verbatim quotes within the applicable cell, retaining the original feel of the transcript. We analysed the matrix to identify mechanisms for changes in travel and to describe the contexts that facilitated or inhibited these changes.

## Results

### Participants

**Fig. 1:**
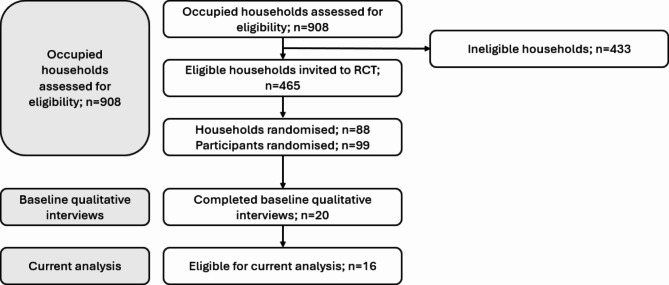
Participant flow diagram

Figure 1 displays participant recruitment in the context of the RCT. Of the 99 participants in the RCT, 20 of the invited subset completed baseline interviews. 16 participants completed at least one follow-up and were eligible for this analysis. Ten participants completed three interviews and six participants completed two interviews, making a total of 42 interviews included in this analysis (Baseline, *n* = 16; 3-month, *n* = 15; 6-month, *n* = 11). Table 2 presents demographic details of interview participants collected at baseline.

**Table 2:** Baseline demographic data

		*n* (%)					
		Qualitative subsample			RCT sample		
**Age**							
	16–24	1		(6)	5		(5)
	25–34	6		(38)	38		(38)
	35–44	3		(19)	34		(34)
	45–54	3		(19)	15		(15)
	55+	3		(19)	6		(6)
**Sex**							
	Male	7		(44)	41		(41)
	Female	8		(50)	53		(54)
	Prefer not to say	1		(6)	5		(5)
**Length of time lived in Northstowe**							
	< 1 month	2		(13)	5		(5)
	2–5 months	4		(25)	12		(12)
	6–12 months	4		(25)	24		(24)
	> 12 months	6		(38)	58		(59)
**Highest level of education**							
	Degree or equivalent and higher	10		(63)	71		(72)
	Secondary school education	5		(31)	21		(21)
	Other	1		(6)	7		(7)
**Housing tenure**							
	Rent property	1		(6)	9		(9)
	Shared ownership	3		(19)	10		(10)
	Privately owned	12		(75)	80		(81)
**Household car ownership**							
	0 cars	2		(13)	13		(13)
	1 cars	10		(62)	42		(42)
	> 2 cars	4		(25)	44		(44)
**Travel to work using car in last four weeks**							
	Never or rarely	5		(31)	40		(40)
	Occasionally	3		(19)	15		(15)
	Usually	1		(6)	12		(12)
	Always	7		(44)	32		(32)

### Summary of common mechanisms identified

We identified six common mechanisms that facilitated modal shift in response to a diverse range of key events. We take each of these mechanisms in turn and describe the key events that led to a change in contexts and the individual and environmental contexts that influenced whether a change in travel mode change occurred. Table 3 describes the generalisable configurations identified. Using an illustrative example, when a bus cancellation (key event) occurs in a context where (i) an individual had planned to use the bus to commute, (ii) they are not able to work from home and are required to be at work on time (for example, a teacher) and (iii) they have access to a car, this leads to a modal shift from bus to car (outcome). The reasoning (mechanism) is that access to the planned transport mode has been removed and the individual is unable to reach the destination on time, leading to a deliberation process and the selection of an alternative mode.

**Table 3:** Generalisable configurations

The key event…	… in this context…	… leads to changes in reasoning… (mechanism)	… and produces this outcome.
**Changes access**			
Reduces access to the planned transport mode	Planned journey is necessary; Acceptable alternative travel mode available	Planned transport mode unable to reach my destination	Modal shift
	Planned journey is necessary; No acceptable alternative travel mode available	Planned transport mode unable to reach my destination	Implementation of coping strategies
	Planned journey is not necessary	Don’t need to make this journey	No travel
Increases access to other transport modes	New destination accessible by alternative mode; Favourable attitudes towards alternative travel mode	Alternative travel opportunities are possible for journey	Modal shift
	New destination accessible only by existing travel mode	No possible alternative available	No modal shift
**Changes reliability**			
Reduces reliability of transport mode due to ongoing events removing access	Reduced reliability experienced on existing transport mode; Planned journey is time dependent; Acceptable alternative travel mode available	No longer able to rely on this transport mode to arrive at destination on time	Modal shift
	Reduced reliability experienced on existing transport mode; Planned journey is time dependent; No acceptable alternative travel mode available	No longer able to rely on this transport mode to arrive at destination on time	Explore modal shift opportunities
Improves reliability of transport mode	Aware of reliability improvements; Preference for transport mode that experienced reliability improvements	Transportation is more reliable and now able to arrive at destination on time	Modal shift
**Changes financial cost**			
Increases financial cost	Planned journey is necessary; Cheaper alternative travel mode is available	Cost of current travel is becoming too expensive	Modal shift
	Planned journey is necessary; Already travelling using the cheapest method	Cost of current travel is becoming too expensive	Continue with current travel mode
	Planned journeys are discretionary	Cost of current travel is too expensive	Reduce journey frequency
Reduces financial cost	Individual actively seeking cheaper alternative; Cheaper alternative is suitable	Cost of current travel is too expensive and a cheaper alternative is available	Modal shift
	Currently travelling on travel mode experiencing reduced cost	Reduced cost is welcome	Continue with current travel mode
**Convenience**			
Requires travel modes that meet demands of dependents	Alternative travel mode provides increased convenience; Responsibility for dependent falls on individual		Modal shift
**Increases confidence**			
Increases confidence	Uncertain or nervous about alternative travel mode; Social support available; Equipment available	Presence of a friend provides re-assurance and a positive experience make future journeys using that mode more likely	Modal shift
**Increases awareness**			
Raises awareness of pro-environmental behaviour	Short journeys replaceable by active transport; Time available account for additional journey time	Current travel mode does not align with view on environmental impact of travel	Modal shift

### Changing access

#### Reduced access

When access to a transport mode was reduced or removed and the planned mode was unable to transport participants to their destination, modal shift was dependent on available alternative modes and characteristics of the planned journey. The key event that reduced access to the existing travel mode triggered modal shift in contexts where the journey was necessary and there was an acceptable alternative mode available. For example, ‘*Yesterday I was not allowed on the bus going into work in the morning and I wasn’t the only one*,* there were about 15 or 20 other people who couldn’t get on the bus because it was full*,* and where they’ve changed the timetable*,* the next bus isn’t for another half an hour after that*,* so I wouldn’t have got to work on time*,* so I had to drive (P0811)’.* We observed examples of events that reduced access to the bus through acute cancellations, occurring due to timetable changes, driver strikes and adverse weather; and that reduced access to the car when ‘*my car was having its service done (P0371)’*, participants had been drinking alcohol, or car parking was unavailable at the destination.

In contexts where travel was required but there was no acceptable alternative mode available, participants implemented alternative strategies which included staying overnight at the destination, changing travel route or time, or taking annual leave because it was not possible to get to the workplace: ‘*I used to be a 9 to 5.30 person. Since using the [local bus service] I’ve changed to being an 8 to 4.30 person just to avoid the rush hour traffic (P3291)’.* This was evident among people without access to a car who used the bus. Where there was no alternative mode available and the journey was not necessary, participants simply did not travel or, where flexibility allowed, worked from home.

#### Increasing access

Some key events led to increased access to alternative travel modes. These included new jobs or colleagues moving to Northstowe. Modal shift occurred among participants who had new jobs when the new workplace was accessible by an alternative travel mode, generating a new stable context with potential to facilitate longer-term modal shift. In some cases, however, the alternative travel modes described would not have been feasible for everyone (for example, not everyone can realistically run to work). For others, meanwhile, both the original and new work locations were accessible only by car, and despite exploring alternative options no modal shift happened for those participants.

### Changing reliability

#### Reducing reliability

The ongoing reduced access led participants to reason that they were unable to rely on those transport modes for consistent journey times that enabled them to arrive at the destination on time. Within this study, the ongoing bus cancellations and overcrowding was sparked from wider contextual changes influencing the transport infrastructure. Participants reported that Britain’s exit from the European Union, led to HGV driver shortages, resulting in a shift of bus drivers to HGV driving leading to an acute shortage of bus drivers and a reduced bus timetable. Once again, modal shift occurred in contexts where reduced reliability affected the existing travel mode and within the context of journeys that were time dependent, for example, travelling to an airport or repeated journeys such as the commute: ‘*On a number of occasions either the bus hasn’t turned up because it’s been cancelled*,* or I’ve not been allowed on because it’s been too full … I think the last interview was close to the point at which I just stopped using the bus altogether because I couldn’t rely on it (P0811)’.* The repeating nature of journeys exposed individuals to the ongoing unreliability which they viewed as disruptive. It was notable that tolerance towards reduced reliability was variable and affected by weather and sustainability values. For those making journeys infrequently or leisure time journeys that were not time dependent, unreliability did not seem appear to trigger modal shift.

Faced with deterioration of the reliability of the bus service, participants who had no alternative travel mode were unable to immediately switch mode. However, the consequences of unreliable bus travel affected their ability to arrive at work on time: ‘*I usually reach five to ten minutes late … despite all the running I do (P1291)’.* This triggered participants to seek alternative travel modes, such as purchasing bikes and learning to drive for those who were physically and financially capable: ‘*My experience for the past two months have kind of like proven that the busway’s not very reliable with regards to time. So I would still gear towards having at least a car for the household*,* just for the situations when I have to get somewhere on time (P1281).’* One participant’s coping mechanism was to change to a job not in line with their career goals in order to cope with the unreliability of the bus service.

#### Improving reliability

Restoration of the bus timetable resulted in a more reliable bus service which reversed the reasoning described above: ‘*it is now my intention to try and use the bus again going forwards and from that one experience I had it does seem to have made things a little better (P0811)’.* In the case of the bus, improving reliability triggered a modal shift among participants whose travel preference was the bus and who were aware of the improvements. We noted a difference in timing returning to bus travel dependent on sustainability motivations, whereby participants with stronger motivations were more eager to restore bus travel.

### Changing financial cost

#### Increasing financial cost

We found that modal shift occurred when participants were faced with rising travel costs, resulting in this study from rising petrol costs linked to global fuel prices, coupled with increased frequency of office working following lifting of covid-19 restrictions. Participants who changed travel mode reasoned that the cost of their current travel mode was becoming intolerable and sought cheaper alternatives. Modal shift occurred in the context of commuting journeys which were required and where cheaper and acceptable alternatives were available. For example, one participant initiated a car share with a colleague who lived nearby, sacrificing the convenience of being a sole car user to reduce journey costs.

In a context in which individuals believed they were already travelling in the cheapest way, no modal shift occurred, but as one participant noted ‘*I’m so glad I’m car sharing because it’s like £10 more expensive to fill up my car (P1191)’.* Furthermore discretionary journeys, for example to eat out at restaurants, did not appear to be subject to modal shift. Instead, participants chose not to make these journeys.

#### Reducing financial cost

We identified one event that reduced the financial cost of travel: ‘*They’ve [bus company] introduced some flexible fares that mean it’s very economical*,* it works out at £2.94 a day for bus travel*,* which is definitely cheaper than petrol (P0811)’.* When this occurred simultaneously with increased commuting frequency described above, the cost difference was amplified and the participant reasoned that bus travel was the cheapest mode and modal shift occurred from the car to the bus. In this context, the participant was actively seeking alternative travel modes due to the cost of using the car. We saw no instances of modal shift due to the reduced bus fares among participants who were unable to use the bus for commuting. Among participants already using the bus, the reduced cost was welcome and while no modal shift occurred, the reduced cost may have contributed to maintaining that travel mode.

### Changing convenience

#### Convenience to meet changing demands of dependents

We noted instances where the key event led to changes in the convenience required of travel due to the demands of dependents. Where this required greater convenience (e.g. the acquisition of a new pet) and an alternative mode provided greater convenience, modal shift occurred. For example, shifting from car-sharing to sole car use enhanced the flexibility to leave work early, or switching from walking to car use decreased the journey time. In contrast, when the key event reduced the need for convenient travel – because a participant’s child was starting school near home, and no longer travelled to with her to the workplace nursery – intentions were formed to travel by bus.

### Increasing confidence

We found that prompts to encourage individuals to try alternative travel modes, including suggestions from friends – ‘*She [friend] said*,* do you want to walk or shall we cycle? So I said*,* no*,* let’s cycle (P2591)’* – or workplace initiatives, resulted in a modal shift towards the bicycle. This shift happened among nervous cyclists when there was social support in place and cycling equipment was available in the wider environment, via spare bicycles or local cycle hire schemes. The social support increased participant confidence that they were capable of cycling. When similar prompts were experienced by nervous cyclists without social support, modal shift did not occur and the participant did not trial cycling, despite the availability of cycling equipment in the local environment.

### Raising awareness

#### Raising awareness of pro-environmental behaviour

Finally, we observed modal shift from the car to walking or cycling for journeys to complete short errands (e.g. posting letters, visiting doctors) due to an enhanced awareness of the environmental impacts of car use and the health benefits of active travel. ‘*I don’t use the car quite so much. I’m either not going anywhere or I’m walking … so I do try and not use it . .because it’s better for me to just to walk everywhere* (P2691)’. These participants reasoned that their current car use did not align with their view on environmental impact, and therefore changed mode. Both participants had sufficient time to complete these journeys, and the proximity of the errands to their homes enabled these journeys to be completed via walking or cycling.

### Common principles

Across the six mechanisms, we identified three notable commonalities. Firstly, when key events disrupted existing travel modes, they often led to changes in travel modes because participants were required to consider alternative travel modes. For example, two participants reported changing their travel mode when their car was in the garage, reflecting a disruption in their current patterns of car use for commuting or errands. In contrast, when events acted on travel modes not currently used by participants they had little influence on their travel choices. For example, deterioration of the bus service was not reported to influence car users’ choice of travel mode. Secondly, we identified the availability of alternative modes and the journey characteristics as important contextual influences. Changes in travel mode were only observed where there was a suitable alternative mode available. In the absence of a suitable alternative participants continued their current travel patterns, although we did observe them forming intentions to increase the availability of alternative modes, by obtaining a driving license or purchasing a bike. We identified the characteristics of the journey as an important context and participants’ travel choice was different dependent on whether a journey was discretionary, necessary or time-dependent. Thirdly, we observed that the experience of the new travel mode played an important role and positive or negative experiences of the alternative mode informed future travel intentions. For example, one participant described a pleasant experience of cycling after commuting as part of a workplace sustainability day, citing the sunset and social aspect as enjoyable aspects that prompted intentions to continue with this mode.

## Discussion

### Summary of findings

We identified six mechanisms, potentially generalisable as intervention strategies, that explained the process of how changes in travel mode occurred: changing access, reliability, financial cost, convenience, confidence and awareness. For some mechanisms we found evidence that they could operate in either a positive or a negative direction, for example increasing and reducing access. We found that these mechanisms were triggered by a diverse set of key events, and we identified the features that may be generalisable to apply as intervention strategies. Firstly, key events acting on existing travel modes appeared to have a greater influence than those acting on potential alternative modes. Secondly, modal shift was only observed among participants who had a suitable and acceptable alternative for the journey, and for whom the journey was not discretionary. Individuals without alternative travel modes available may have to implement coping strategies, for example starting work at a different time or taking annual leave if their existing transport mode is disrupted. Thirdly, the experience of the alternative travel mode appeared to influence future intentions.

### Comparison to previous literature

Similar to the findings from this study, previous reviews of interventions have identified that financial mechanisms appear to be effective for reducing driving behaviour [22], and mechanisms involving accessibility, awareness and experience appear to be effective for increasing active travel [22, 23]. The fact that we identified these mechanisms suggests commonalities in the mechanisms of travel behaviour change, regardless of whether the event originates from deliberate interventions or from other sources. Previous research has identified additional mechanisms relating to aesthetics, safety, skills and space [22]. These may be more likely to originate from interventions or environmental changes with a deliberate intention to change travel behaviour, and are unlikely to have occurred in Northstowe during the six months of our study. We have previously identified that the same event might have different mechanisms depending on its context, and that within some contexts these mechanisms may not be triggered at all [23]. Those principles are reinforced by the current study, in which we identified that individuals responded differently to the same events depending on characteristics of the journey and the availability of alternative travel modes. This analysis therefore contributes to an emerging field of research focusing on understanding the process of how travel behaviour change occurs.

Many of the concepts identified in this analysis are consistent with other conceptualisations or framings. We identified mechanisms operating in both positive (reducing cost or increasing confidence) and negative directions (reducing access, increasing cost or reducing reliability), described elsewhere as ‘carrots’ or ‘sticks’ respectively [24]. We found no evidence of a negative direction for the mechanisms of experience, awareness and convenience in this study, but it is plausible, for example, a change in bus route may reduce the convenience of the bus. A previous review found that interventions using carrot and stick strategies in combination, or sticks alone, were more effective than interventions using only carrot strategies [22]. For example, workplace travel plans including parking restrictions and charging (sticks) plus off-site parking provision and improved bus services (carrots) were more effective than strategies to encourage alternatives to driving (carrot) alone [25]. Our study gleans additional insight, suggesting that ‘sticks’ often disrupted existing travel modes. We found that key events that operate as ‘carrots’ often act on travel modes not currently used, and may therefore be more influential among those predisposed to walking and cycling but insufficient to spur broader modal shift among those without such dispositions [24]. The habit discontinuity hypothesis supports these insights, whereby disruptions to a stable context lead to active deliberation about travel mode [26]. Research in this domain suggests that strong habitual tendencies attenuate information acquisition whereby those with strong travel habits are less likely to notice and acquire information about alternative travel modes [26], potentially explaining why few participants noted key events that affected alternative travel modes. Furthermore, interventions that require individuals to use a high levels of personal resources (or agency) in order to benefit from the intervention are hypothesised to be less effective and equitable, compared to those with low demands [27]. Exposure to key events that impact on existing travel modes require individuals to use few or no personal resources compared to those operating on alternative travel modes [28], suggesting that they are more likely to trigger deliberation. Taken together, this suggests that an event disrupting existing travel behaviour, and acceptable alternatives may be required to ensure such policies are equitable.

Implementing more disruptive ‘stick’ approaches poses challenges for public and political acceptability [29], due to the perceived political risk that has potential to instigate policy conflict or public backlash against future policies [24]. However, implementing these alongside positive and supportive ‘carrot’ approaches that provide acceptable alternatives to facilitate travel behaviour change has the potential to enhance their acceptability. The different disciplinary theories described above describes commonalities and synergies, and bringing them together contributes to ‘*holistic sense-making’* and strengthens the generalisability of these findings across behaviours, contexts and populations which can inform future interventions [15]. 

### Implications for intervention research

Understanding how changes in travel mode occur in response to a variety of events can help to inform intervention strategies. Key events that act on existing travel modes, particularly those that operate to deter their use, appear to influence modal shift. Applying this principle to interventions suggests strategies to reduce access, reduce reliability and increase financial cost of car use to facilitate modal shift towards active travel and public transport. These mechanisms could be triggered by interventions such as road user charging, pedestrian zones or speed restrictions. However, a cautious approach is required because interventions of this kind are often less publicly and politically acceptable [29], and ensuring that walking, cycling and public transport infrastructure provides and accessible, convenient and pleasant alternative is likely to be a pre-requisite to enhance their effectiveness, equity and acceptability [30]. Without this, we found that individuals are likely to continue using their existing transport mode with barriers in place by implementing coping strategies, such as changing jobs, taking annual leave, changing working hours or being late to work. This may have important implications for some population groups and these should be identified to assess the equity impacts of interventions and to explore potential unintended consequences, for example on wellbeing or employment.

We also found that key events acting on transport modes not currently used by participants were described as leading to travel behaviour change among participants already considering those travel modes, suggesting that this approach may have limited success for attracting new walkers, cyclists and public transport users. Furthermore, we observed several instances of one-off travel behaviour change and found that these presented opportunities to form opinions on alternative travel modes. In order to capitalise on such chance opportunities, efforts should be made to ensure that experiences are pleasant, reliable and accessible to increase the likelihood of future modal shift [31]. 

### Strengths and limitations

A strength of the study is its longitudinal design to assess temporal travel behaviour trends. The follow-up period was sufficient to report on intentions for travel behaviour change and allowed sufficient time for changes to occur, while being short enough to be unlikely to impact recall. We purposively sampled participants according to their car ownership and travel patterns to understand experiences from a range of participants with differing initial travel patterns. This analysis is based on interviews collected as part of an RCT exploring financial incentives to reduce car use, and therefore participants may have self-selected to participate in a travel related study. Further selection bias is possible whereby participants in the semi-structured interviews may have been more interested in environmental and health issues. Despite little evidence that these incentives affected travel behaviour [15], participation in the study may have subliminally primed individuals to consider or change their travel behaviour. Furthermore, our reliance on information provided by participants may have not have identified additional mechanisms that operate via less deliberative process of reasoning. Participants in this study reflect a relatively affluent population [18], and it is unclear whether key events would trigger the same functions in different population groups, or whether the availability of alternative travel modes differs by population group.

## Conclusion

In this study we applied realist evaluation principles to understanding travel behaviour change. We identified six common mechanisms that facilitate travel behaviour change: increasing or reducing access, reliability and financial cost, improving convenience and confidence, and raising awareness. We observed changes in travel behaviour when these mechanisms disrupted an existing travel mode and when an alternative travel mode was available. This study contributes to understanding how key events change travel behaviour and provides evidence about what contexts appear supportive of change. The mechanisms identified here could form targets for intervention strategies and could be generalisable to a range of other settings.

### Electronic supplementary material

Below is the link to the electronic supplementary material.

Supplementary Material 1

## Data Availability

The datasets analysed during the current study are not publicly available as it is not possible to fully anonymise the interview transcripts.
