# Supplementary material for: Understanding how and why travel mode changes: analysis of longitudinal qualitative interviews
Source: Int J Behav Nutr Phys Act. 2024 Sep 2;21:96. doi: 10.1186/s12966-024-01647-x (PMC11367882; doi:10.1186/s12966-024-01647-x)
Supplement: Supplementary file 1 — Supplementary Material 1 [file 12966_2024_1647_MOESM1_ESM.docx]

**Interview Topic Guide**

**Interview 1;** Assess experiences of moving to Northstowe, how travel patterns were established and initial impressions of the financial incentives

**Interview 2;** Assess ongoing use of financial incentives

**Interview 3;** Assess ongoing use of financial incentives; explore alternative financial incentives that might influence active travel

**1. Introduction**Explain purpose of research project
Explain audio recording procedures
Ensure participant has a copy of latest participant information sheet
Answer any questions
Check e-consent has been completed
Commence audio recording

**2. Experience of moving home**

When did you move to Northstowe?

Why did you want to move? (buying participants)

Why were you offered a move? (social housing)

What were you hoping for from your move?

What are your views about your neighbourhood? Physical/social environment?

What has been good about moving? What’s been less good?

Prompts:

Cost of home

Sustainability

Future vision for Northstowe

Availability of homes

Location (proximity to work/family/activities/schools)

Feelings of community cohesion/developing new community

Working patterns

**3. Travel in Northstowe**

How do you mostly get around? Is that the same or different to how you travelled before you moved?

Has the way you travelled changed throughout your time here?

Have you tried other methods of transport?

If applicable, is that the same or different to how you travelled before the pandemic?

How have you adapted to the new physical environment of Northstowe?

Within the Northstowe development, what are your experiences of travelling?

Journeys made in a typical day? Work? School? Errands?

How do other members of your household travel?

**4. Typical day**

In terms of travel, what does a typical day look like for you?

How is this different on a weekend?

Origin and destination of journey
Usual route followed and (combination of) mode(s) of transport used

Variation on usual route/which factors determine what route they take

Whether these have changed or have/have plans to experiment with other modes (combinations?)

Prompts:

Time of day

Distance/time spent travelling

Weekend vs weekday

Leisure activities vs work/errands

5**. Reasons for these choices and alternatives available**

Why choose this (combination of) mode(s)/routes?

Could you make that using alternative modes of travel?
Could the journey be made by other routes or other (combinations of) modes?
What factors influence the choice between these modes?

Which of these are most important, for example if you could only choose one? (Repeat reasons back clearly to participants)

Prompts to be used if necessary:

| Availability of other modes | Habit |
| --- | --- |
| Comfort | Need to carry passengers or luggage |
| Convenience | Privacy |
| Cost | Safety |
| Distance | Time |
| Environmental concerns | Trip-chaining |
| Exercise | Weather |

**6. Variations on the typical journey and reasons for those variations**

Prompts to be used if necessary:

When starting or finishing work at different times

When a particular mode of transport (e.g. family car) is (not) available

When transporting children or other passengers, shopping, trip-chaining

Weather conditions

**7. Potential for change, barriers and facilitators**

Any expectation or intention of changing travel mode(s)?
What factors act as barriers to making that change?
What factors would facilitate that change?
Why do you think other commuters make other travel choices?

Prompts listed above under 5 to be used as necessary

| Availability of other modes | Habit |
| --- | --- |
| Comfort | Need to carry passengers or luggage |
| Convenience | Privacy |
| Cost | Safety |
| Distance | Time |
| Environmental concerns | Trip-chaining |
| Exercise | Weather |
| Sustainability |  |

**8. Claiming financial incentives (all participants)**

When you moved in, why didn’t you take the incentives on offer?

What were the barriers?

Are there any changes to the process that would mean you would have claimed them?

What types of financial incentives do you think might encourage other people to change travel behaviour?

What types of financial incentives do you think might encourage you to change travel behaviour?

**9a. Group 1: Plans to claim incentives**

As you are allocated to the control group and will need to claim these directly, do you intend to claim any of the vouchers?

Which vouchers do you intend to claim?

Why will you claim [insert voucher type]?

Why don’t you plan to claim [insert voucher type]?

How do you anticipate these will influence your travel behaviour?

**9b. Group 2&3: Initial impressions use of financial incentives**

Check they have received and read? Skim read/thorough read?

What is your understand of what vouchers on offer and how you might claim them?

What are your initial impressions of the incentives provided?

Have you used any yet? If so, how have you used them? (Specific for person who has used each)

Do you intend to use any of these incentives? If yes, how do you anticipate you’ll use them?

Are there any barriers to using these incentives?

Prompts:

Awareness of financial incentives

Household distribution

Contamination

Continuation of behaviour after vouchers

Decathlon

Cycle maintenance

Greenbelt

E-Bike

Stagecoach

**10. Close**

End audio recording
Thank for participating
